# Knowledge and performance of the Ethiopian health extension workers on antenatal and delivery care: a cross-sectional study

**DOI:** 10.1186/1478-4491-10-44

**Published:** 2012-11-21

**Authors:** Araya Medhanyie, Mark Spigt, GeertJan Dinant, Roman Blanco

**Affiliations:** 1 Department of Public Health, College of Health Sciences, Mekelle University, Mekelle, Ethiopia; 2 CAPHRI, School for Public Health and Primary Care, Maastricht University, Maastricht, Netherlands; 3 Department of Medicine, University of Alcala de Henares, Madrid, Spain; 4 Department of General Practice, Tromso University, Tromso, Norway

**Keywords:** Community health workers, Health extension workers, Antenatal care, Primary health care, Maternal health care

## Abstract

**Background:**

In recognition of the critical shortage of human resources within health services, community health workers have been trained and deployed to provide primary health care in developing countries. However, very few studies have investigated whether these health workers can provide good quality of care. This study investigated the knowledge and performance of health extension workers (HEWs) on antenatal and delivery care. The study also explored the barriers and facilitators for HEWs in the provision of maternal health care.

**Methods:**

In conducting this research, a cross-sectional study was performed. A total of 50 HEWs working in 39 health posts, covering a population of approximately 195,000 people, were interviewed. Descriptive statistics was used and a composite score of knowledge of HEWs was made and interpreted based on the Ethiopian education scoring system.

**Results:**

Almost half of the respondents had at least 5 years of work experience as a HEW. More than half (27 (54%)) of the HEWs had poor knowledge on contents of antenatal care counseling, and the majority (44 (88%)) had poor knowledge on danger symptoms, danger signs, and complications in pregnancy. Health posts, which are the operational units for HEWs, did not have basic infrastructures like water supply, electricity, and waiting rooms for women in labor. On average within 6 months, a HEW assisted in 5.8 births. Only a few births (10%) were assisted at the health posts, the majority (82%) were assisted at home and only 20% of HEWs received professional assistance from a midwife.

**Conclusion:**

Considering the poor knowledge of HEWs, poorly equipped health posts, and poor referral systems, it is difficult for HEWs to play a key role in improving health facility deliveries, skilled birth attendance, and on-time referral through early identification of danger signs. Hence, there is an urgent need to design appropriate strategies to improve the performance of HEWs by enhancing their knowledge and competencies, while creating appropriate working conditions.

## Background

It is estimated around 358,000 maternal deaths from complications of pregnancy and child birth occurred worldwide in 2008. Of these, developing countries accounted for 99% (355,000) of deaths [1]. The vast majority of maternal deaths are due to direct obstetrical complications, including hemorrhage, infection, eclampsia, obstructed labor, and unsafe abortion. Most obstetric complications occur around the time of delivery and cannot be predicted, but can be prevented with proper medical care [2]. In 2008, Ethiopia was among the six countries that contributed for more than 50% of maternal deaths in the world [3]. There is no evidence suggesting maternal mortality in Ethiopia is declining. In Ethiopia, 673 and 676 maternal deaths occurred in every 100,000 live births in 2005 and 2011, respectively [4,5].

Ethiopia is a signatory to the millennium development goals. Goal 5 targets the reduction of maternal mortality by 75% between 1990 and 2015 and calls for a target of 80% of births assisted by a skilled attendant by the year 2015 [6]. Skilled birth attendance is advocated as the ‘single most important factor in preventing maternal deaths’ and the ‘proportion of births attended by skilled health personnel’ is one of the indicators for millennium development goal 5 [6,7]. The World Health Organization (WHO) defines a skilled birth attendant as ‘an accredited health professional - such as a midwife, doctor or nurse - who has been educated and trained to be qualified in the skills needed to manage normal (uncomplicated) pregnancies, childbirth and the immediate postnatal period, and in the identification, management and referral of complications in women and newborns’ [7].

Since the Alma-Ata Declaration on primary health care in 1978, community health worker cadres have been occurring in many countries across the globe [8]. Nowadays, the potential roles of community health workers within primary health care have received renewed attention as a result of the HIV pandemic and due to the increasing acknowledgement of the critical shortage of human resources within health services [9-11].

Similar to other developing countries, Ethiopia has been training and deploying volunteer community health workers. With the aim of accelerating primary health care coverage and ensuring access to basic health services to the underserved rural population, the country launched a new community-based initiative called the Health Extension Program (HEP) in the year 2003 [12]. Under the umbrella of this program, cadres of community level health workers trained for 1 year, named health extension workers (HEWs), were deployed to rural areas. In relation to reducing maternal mortality, HEWs are trained on how to provide care to pregnant mothers through pregnancy, birth, and postnatal care. They are part of the formal health structure and receive a monthly salary.

Very few studies have been published on the effectiveness of these HEWs since their deployment [13]. These studies have shown that HEWs are effective in improving immunization, family planning utilization, and antenatal care visits, but not in health facility deliveries and skilled birth attendance coverage [13-15]. Reviews have been published concerning the role of community health workers, highlighting successes and problems in other developing countries [16-19]. A systematic review done to assess the effectiveness of lay health workers showed promising benefits in promoting immunization uptake and improving outcomes for acute respiratory infections and malaria. However for other health issues such as birth attendance, there is insufficient evidence to justify recommendations which can guide policies and practices [10].

Considering that HEWs are not skilled birth attendants, it is recommended that the HEWs task should focus on the early identification of danger signs, danger symptoms, and complications in pregnancy and facilitating immediate referrals of pregnant women when needed. However, it is not known whether the knowledge of HEWs is adequate. Therefore, we examined the knowledge of HEWs on contents of antenatal care, danger signs, danger symptoms, and complications in three districts of Tigray region in Northern Ethiopia. In addition, we explored barriers and facilitators for HEWs in the provision of antenatal care, delivery care, and referral services.

## Methods

### Study design

The study employed a descriptive cross-sectional design.

### Setting

The study was carried out in three selected districts of Tigray region, Ethiopia; namely Kilte Awlaelo, Saesi Tsadamba, and Degua Temben districts. Tigray is the most northern regional state of Ethiopia. The total population of the region was 4.6 million in 2010 [20].

The Ethiopian health system is a four-level health system, characterized by the lowest level called a primary health care unit, comprising one health center and five satellite health posts, and then the district hospital, zonal hospital, and specialized hospital [12]. Health centers are staffed with a health professionals’ team, including midlevel health professionals, for instance, health officers, nurses, midwives, sanitarians, and laboratory technicians. Ideally, there are five satellite health posts under one health center which means one health center supervises and receives referrals from five satellite health posts. Health posts are the operational units for HEWs.

### Participants and sampling

Since all HEWs and health posts in the three selected districts were eligible for the study, we did not use any specific sampling technique. Rather, the focus was at ensuring participation of as many HEWs as possible. To do this we had the list of all kebeles in the districts and HEWs working in each kebele. A kebele is the smallest administrative unit in Ethiopia. It is synonymous with a village which has an average population of 5,000 people. After getting permission from Tigray regional health bureau to undertake the study, the data were then collected by travelling to each kebele to meet the HEWs for an interview.

### Data collection

Initially a semi-structured interview type questionnaire on paper was prepared by reviewing guidelines and manuals for HEWs. The questionnaire was divided into four sections. Section 1 was on the bio-data and characteristics of HEWs. Section 2 dealt with the availability of supplies, facilities, and logistics at health posts for maternal health care. This availability of supplies, facilities, and logistics was confirmed by observation. Section 3 was about knowledge and performance of HEWs on contents of antenatal care, birth assistance, danger symptoms, danger signs, and complications in pregnancy and making referrals. Section 4 was about barriers and facilitators for HEWs in maternal health services provision.

This hard copy questionnaire was then converted to an online questionnaire using software called Episurveyor and downloaded to a mobile phone (Nokia E71) [21]. Episurveyor is a web-based system which allows users to create a questionnaire online, download the questionnaire to a mobile phone, fill out the questionnaire using the phone, send it to a server, view data online, and export data into statistical software for further analysis. Downloading an online created questionnaire on a mobile phone was possible directly through an internet connection at the mobile phone or downloading the online questionnaire first to a personal computer (PC) and then transferring it to a phone using Universal Serial Bus (USB). All the data collected can also be saved in the memory of the mobile phone and backed up to a remote server, where it can be analyzed later.

Prior to the actual data collection, the online questionnaire downloaded on the mobile phone and using mobile phone for interview were pre-tested. The pre-test was done to assess clarity of the questions, time needed to finish the interview, and to know respondents’ comfort to be interviewed using a questionnaire on a mobile phone. This pre-test was done by interviewing five HEWs who were not included in the actual study. One major finding of the pre-test was that few of the questions had a long list of options and were found to be time-consuming when asked using mobile phones. The interviewer had to scroll down and up several times to fill out the responses of respondents. Hence, we decided to exclude these few questions from the online questionnaire and included them on paper instead. All the interviews were conducted by the principal investigator. We chose the principal investigator; because new data collectors may not have been familiar with the mobile phone approach of data collection, and we had an interest to understand very well whether mobile phone data collection can be feasible for subsequent studies in the Ethiopian context.

### Data analysis

The collected data which was submitted to the database server was exported to SPSS version 16 (SPSS Inc, Chicago, IL, USA) for analysis. Descriptive statistics was used to summarize the data and the results were presented using frequency tables and percentages. To assess the knowledge of HEWs on contents of antenatal care counseling, danger symptoms, danger signs, and complications in pregnancy, relevant questions from the questionnaire had weights attached to them to create a composite score of knowledge. For the knowledge on contents of antenatal care counseling service, the maximum score was 25 points and points were awarded on a discrete (whole number) rather than a continuous scale, based on the number of positive responses. Interpretation of scores was based on the Ethiopian university education scoring system. We used this scoring system, because we could not find a standard scoring system for evaluating the HEWs’ knowledge. Hence, we used the Ethiopian university scoring system by slightly modifying it into a four-scale ranking. Respondents whose scores were 80% or more were classified as having excellent knowledge on contents of antenatal care counseling; those who scored between 60% and 79% were classified to have good knowledge; those who scored between 45% and 60% were classified to have fair knowledge; and those who scored 45% and below were classified as having poor knowledge. We slightly modified the Ethiopian university scoring system into a four-scale ranking for convenience because the Ethiopian scoring system has a long list of levels. A similar approach was used to interpret the knowledge of HEWs on danger symptoms, danger signs, and complications in pregnancy. Additional interpretations of scores were also made using the mean values of respondents’ knowledge. The mean and median values for the knowledge scores were virtually the same.

### Ethical consideration

The study was approved by an ethical review committee at the Tigray regional health bureau in Ethiopia. The purpose of the study and the use of mobile phones were explained to each respondent. A verbal consent to participate in the study was obtained from each respondent. Participants were also informed about their right to withdraw from the study at any time of the data collection if they felt any discomfort.

## Results

### Characteristics of HEWs

A total of 50 out of 68 HEWs working in 39 health posts which cover a population of approximately 195,000 people were interviewed. The remaining 18 (26.5%) were not present in their working place or kebele during the period of data collection for different reasons such as meetings, training, maternity leave, or social reasons. All respondents were women and their age ranged from 22 to 38 and the mean age was 26.36 (SD: ±4). Thirty-six (72%) of them were married. Almost half (48%) of them had at least 5 years of work experience as a HEW.

### Performance of HEWs in assisting births and referrals

Eighty-two percent of the HEWs received additional on job training on antenatal care, and clean and safe delivery. Ninety-two percent of the HEWs had been assisting in births within 6 months prior to the data collection (Table 1). Within 6 months, a HEW assisted in 5.8 births on average. Only a few births (10%) were assisted at the health posts, the majority (82%) were assisted at home. HEWs rarely referred to a health center as is shown by the low percentages of HEWs who had made such a referral. About 48% of the HEWs made a referral to health center during antenatal care, while 54% of them made referrals during labor and delivery. In addition, receiving professional assistance from midwives on obstetric care was rare. Only 20% of the HEWs had received professional assistance from a midwife.

**Table 1:** **Characteristics and performance of HEWs in assisting births and referral services (*n*=50)**

**Characteristics and performance**	**%**
Years of working experience as HEW	
1 to 2	36
3 to 4	16
5 or more	48
HEWs with mobile phones	92
HEWs who received additional on job training on antenatal care, clean and safe delivery at least once	82
Performance of HEWs within 6 months prior to data collection	
HEWs who made a referral of pregnant woman at least once during antenatal care visits to health center	48
HEWs who made a referral of pregnant woman during labor or child birth to health center	54
HEWs who received professional assistance related to antenatal care or birth care from midwives at least once	20
HEWs who assisted at least one birth	92
Number of births assisted by HEWs ( mean, 5.82; median, 4.00)	
0	4
1 to 5	28
6 to 10	12
11 to 15	5
16 or more	1
Place of births at which HEWs assisted in births prior to data collection	
Did not assist	8
Health post	10
Home	82

### Characteristics of health posts

More than 85% of the health posts had a vaccine carrier, syringes and needles, functional blood pressure apparatus, functional thermometer, delivery kit, delivery couch, and functional fetoscope (Table 2). Nevertheless, many of the health posts did not have basic infrastructures such as electricity, water supply, and a fixed telephone (only available in 8%, 5%, and 21% of the health posts, respectively). Moreover, none of the health posts had any protocols to aid HEWs in decision-making related to maternal health care.

**Table 2:** **Availability of facilities, supplies, and equipment at health posts (*n*=39)**

**Health posts with**	**%**
Functional fetoscope	100
Delivery kit	97
Vaccine carrier with at least four ice packs	97
Delivery couch	95
Functional thermometer	95
Functional blood pressure measuring apparatus	92
Misoprostol	90
Adequate syringes and needles, gloves	87
Log book	87
Anti-malaria drugs (Coartem)	72
Functional weighing scale	69
Antiseptics, alcohol, and savlon	59
Iron tablets	51
Fixed telephone	21
Safe water supply	5
Electricity	8
Protocols to aid HEWs for decision-making in antenatal care, delivery, postnatal care, and referral	0

### Knowledge of HEWs on contents of antenatal care counseling

The knowledge of HEWs concerning the contents of antenatal care counseling was poor. On average, a HEW knew 11 out of the 25 contents of ANC counseling asked during the interview. Only one respondent (2%) mentioned more than 80% of the 25 contents, nine of the respondents (18%) had good knowledge, 13 (26%) had fair knowledge, and 27 (54%) had poor knowledge. The contents of antenatal care counseling that were usually known and discussed by HEWs with clients were the importance of institutional delivery (86%), taking extra amounts of food (86%), and taking iron folate (80%). Out of the 25 contents of antenatal care counseling included in our survey, 14 of them had been known and discussed with clients by less than half of the HEWs (Table 3).

**Table 3:** **Reported contents of antenatal care counseling known and discussed by HEWs to client (*n*=50)**

**Contents of antenatal care counseling discussed**	**%**
Importance of institutional delivery	86
To take extra amounts of food	86
Give information about HIV/AIDS	82
Take iron folate tablets	80
Counsel on birth preparedness	76
Expected date of delivery	74
Importance of skilled birth attendant	72
To get checked up during pregnancy	64
To get TT vaccination	56
To save money for emergency	54
To seek care if there is a health problem	52
To keep environmental sanitation and personal hygiene	46
To give colostrum to the baby	46
To avoid heavy work	44
Antenatal care at least four visits	44
Tell about danger signs during pregnancy	40
No pre-lacteals	32
Exclusive breastfeeding	30
To take rest	26
Put the baby to breast immediately after delivery	24
To arrange for emergency transport	18
Delay bathing until after 24 h	18
To sleep under a bed net	14
Nothing to be applied to the umbilical stump	4
Lactational amenorrhea method	0

### Knowledge of HEWs on danger symptoms, danger signs and complications in pregnancy

Similar to the knowledge of HEWs on contents of antenatal care counseling, the general knowledge of HEWs on danger symptoms, danger signs, and complications was poor. On average, a HEW knew nine out of the 24 danger symptoms, danger signs, and complications asked during interview. No respondent received an excellent score; only one (2%) had good knowledge, five (10%) had fair knowledge, and the majority 44 (88%) had poor knowledge. The most commonly known danger sign was vaginal bleeding which was mentioned by 98% of the HEWs while important danger symptoms such as severe headache and visual disturbance were known by less than half of the HEWs (Table 4).

**Table 4:** **Reported danger symptoms, danger signs, and complications of pregnancy known by HEWs (*n*=50)**

**Danger symptoms, signs, or complications**	**%**
Vaginal bleeding	98
Prolonged labor (>24 h)	72
Baby’s hands or feet come first	72
Convulsions	58
Retained placenta	54
Edema	52
Anemia	46
High blood pressure	46
No fetal heartbeat	40
Mal-presentation	38
Severe headache	30
Multi-fetal pregnancy	30
Intrauterine fetal death	28
Severe vomiting	26
Offensive or irritating vaginal discharge	24
High fever	24
Low blood pressure	18
Visual disturbances (blurred vision)	12
Ruptured uterus	12
Prolapsed cord	8
Abdominal pain associated with episodes of fainting	2
Burning epigastric pain	0
Preterm rupture of membrane	0
High pulse rate	0

### Barriers and facilitators for HEWs in provision of antenatal care and delivery service for pregnant women

Lack of behavioral change among community to give birth at health facilities, low utilization of health posts by community, and absence of further education for HEWs were the three major reported barriers in provision of maternal health services as mentioned by 72%, 62%, and 56% of the HEWs, respectively (Table 5). HEWs mentioned the presence of volunteer community health workers, increasing proportion of women who were visiting HEWs or health facilities for antenatal care, and provision of maternity leave for pregnant women from safety net programs to be the main facilitators in provision of maternal health services.

**Table 5:** **Barriers and facilitators for HEWs in provision of antenatal and delivery care (*n*=50)**

**Barriers and facilitators reported by HEWs**	**%**
Barriers mentioned by HEWs	
Lack of behavioral change (lack of awareness and wrong cultural beliefs)	72
Low utilization of health posts by community	62
No further education for HEWs	56
High work load of HEWs	48
Low competency of HEWs	44
Giving too much focus on environmental sanitation and less attention to maternal health care	38
Transportation problem	36
Health posts are less equipped (no water, electricity, waiting rooms, and so on)	34
Long walking distance and topographical problems	32
Low salary for HEWs	26
Less confidence of community on HEWs	16
No residence rooms at the health posts for HEWs	14
Less support for HEWs from kebele leaders	10
Meetings	10
Facilitators mentioned by HEWs	
Presence of volunteer community health workers	62
Increasing proportion of women visiting HEWs or health facilities for antenatal care	60
Maternity leave from safety net program	46
Support from kebele administration	24
Support from supervisors	24
Presence of family health card for providing health education for women	20
Support from other sectors (women’s association/non-governmental organizations/agriculture sector)	20
Availability of supplies at health posts	10
Community mobilization and conversation	8
Presence of ambulance	8

## Discussion

The HEWs of Ethiopia play a rather small role in assisting births. On average, a HEW assisted approximately six births per 6 months. Most deliveries took place at home without the necessary professional help or the necessary facilities. The HEWs knowledge on danger symptoms, danger signs, and complications in pregnancy was poor. In relation to this, it was indicated that HEWs rarely referred a pregnant woman to a health center. Very few HEWs received professional support on obstetric care from a midwife.

Studies [13-15] showed that the deployment of HEWs has improved some aspects of maternal and child health such as family planning utilization, immunization uptake, and the number of antenatal care visits but not in health facility deliveries and skilled birth attendance coverage. Nevertheless, these studies did not explore the reasons for HEWs’ low performance in promoting health facility deliveries and skilled birth attendance. Our study showed that one possible reason for the low performance of HEWs in stimulating behavioral change among the community and facilitation of referrals could be their poor knowledge on contents of antenatal care, danger signs, danger symptoms, and complications in pregnancy. Because of their poor knowledge, HEWs may not convince pregnant women to have birth planning and preparedness to give birth at health facilities and assisted by skilled birth attendant. In addition, the HEWs may experience that the public still prefers to give birth at home; despite the fact that the importance of institutional delivery has been discussed with the clients. This choice might be preferable considering the poor knowledge of the HEWs and lack of basic infrastructures at health posts, but it may be also due to a deep-rooted behavior and preference of the community to give birth at home. Given the HEWs are the key and main provider of primary health care services to the rural community in Ethiopia, improving their competency and effectiveness on maternal health care is urgently needed. A study conducted among community health extension workers in Nigeria showed that community health workers, who were backed by telephone consultations and working under the direct supervision of doctors, can improve quality of care to the satisfaction of most of their patients [16]. The recent introduction of mobile phones could provide new opportunities for two-way communication between front-line health workers such as HEWs and skilled birth attendants such as midwives in health centers [22]. However, further researches are needed to investigate the potential impact of mobile phone-based applications in improving the performance of HEWs.

Looking from the HEWs’ perspective, our study showed the absence of further education to improve their career and knowledge, earning low salary, and work load were noted to be the main factors that hindered the HEWs from providing good quality of care. Therefore, it may be unrealistic to expect HEWs to play a key role in the improvement of maternal health care without addressing their needs in career promotion and other monetary incentives. Similar findings were observed in other studies on similar initiatives [18,19,23]. These studies showed that continuous training, transport means, adequate supervision, and motivation of community health workers through the introduction of financial incentives are among the key factors to improve the work of community health workers. Nevertheless, more studies are needed before we can be sure what the best and most cost-effective strategy is to improve the quality of care provided by the HEWs.

Some limitations of this study deserve attention. Although this study was carried out in rural districts, these districts were relatively near to urban towns. We also did not investigate actual care given by HEWs for example by non-participant observation. Presumably the situation is more severe in very remote areas and a similar study [17] like ours, which included non-participant observation, showed that HEWs performed less well when compared to their reported knowledge. Therefore, although the situation observed in our study was far from ideal, we assume that the knowledge and performance of HEWs might be poorer in reality.

We adapted the Ethiopian university scoring system to interpret HEWs’ knowledge on contents of ANC counseling, danger signs, danger symptoms, and complications in pregnancy. Although it might seem illogical to use the university scoring system for HEWs, it has no influence on the description we made, because basically we adapted the knowledge questions in the assessment from the guidelines, manuals, and log books of HEWs. All the knowledge questions were about the contents of ANC counseling, danger symptoms, danger signs, and complications that are expected to be known by HEWs.

Eighteen (26.5%) of the 68 HEWs who were working in the 39 health posts were not present in their working place or kebele during the period of data collection. They moved away from their working place for meetings, training, maternity leave, or social reasons. Their absence was not because they had different characteristics from the HEWs who were interviewed. Hence their exclusion from our study is not likely to influence the findings in this study.

In this study we explored the barriers and facilitators for HEWs in the provision of maternal health services through a questionnaire. However qualitative assessment either through focus group discussions or in-depth interviews with the HEWs might have been preferable approach to explore these barriers and facilitators. Hence, we recommend further qualitative studies in this regard.

## Conclusion

HEWs knowledge on contents of antenatal care counseling, danger symptoms, danger signs, and complications in pregnancy was poor and there was no good referral system. Hence, there is an urgent need to design appropriate strategies to improve the performance of HEWs by enhancing their knowledge and competencies, while creating favorable working conditions for HEWs in the rural areas.

## Competing interests

The authors declare that they have no competing interests.

## Authors’ contributions

AM contributed to the design, data collection, analysis, and write-up. MS, GJD, and RB contributed to the design, analysis, and write-up. All authors read and approved the final manuscript.
